# Crystal structure of Ba_5_In_4_Sb_6_

**DOI:** 10.1107/S2056989015006933

**Published:** 2015-04-09

**Authors:** Ming-Yan Pan, Sheng-Qing Xia, Xu-Tang Tao

**Affiliations:** a State Key Laboratory of Crystal Materials, Institute of Crystal Materials, Shandong University, Jinan, Shandong 250100, People’s Republic of China

**Keywords:** crystal structure, barium, indium, anti­mony, In—In inter­connections

## Abstract

The title compound, penta­barium tetra­indium hexa­anti­mony, was synthesized by an indium-flux reaction and its structure features layers composed of edge-sharing In_2_Sb_6_ units. The voids between the In_4_Sb_6_ layers are filled by Ba^2+^ cations, which are all surrounded by six Sb atoms and form bicapped octa­hedral or triangular prismatic coordination geometries. There are five barium ions in the asymmetric unit: one has no imposed crystallographic symmetry, two lie on mirror planes and two have *mm*2 point symmetry. The two In atoms and four Sb atoms in the asymmetric unit all lie on general crystallographic positions.

## Related literature   

For geometrical details of In—In inter­connections in other structures, see: Iandelli (1964 ▸); Goforth *et al.*, (2008 ▸).

## Experimental   

### Crystal data  

Ba_5_In_4_Sb_6_
*M* *_r_* = 1876.48
Orthorhombic, 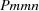

*a* = 14.2723 (13) Å
*b* = 18.3578 (17) Å
*c* = 8.2710 (8) Å
*V* = 2167.1 (4) Å^3^
*Z* = 4
Mo *K*α radiation
μ = 20.39 mm^−1^
*T* = 120 K
0.06 × 0.03 × 0.02 mm

### Data collection  

Bruker APEXII CCD diffractometer
Absorption correction: multi-scan (*SADABS*; Bruker, 2005 ▸) *T*_min_ = 0.374, *T*_max_ = 0.686
12962 measured reflections
2676 independent reflections
2098 reflections with *I* > 2σ(*I*)
*R*_int_ = 0.050

### Refinement  

*R*[*F* ^2^ > 2σ(*F* ^2^)] = 0.029
*wR*(*F* ^2^) = 0.055
*S* = 1.02
2676 reflections
79 parameters
Δρ_max_ = 2.00 e Å^−3^
Δρ_min_ = −1.69 e Å^−3^

### 

Data collection: *APEX2* (Bruker, 2005 ▸); cell refinement: *SAINT* (Bruker, 2005 ▸); data reduction: *SAINT*; program(s) used to solve structure: *SHELXS97* (Sheldrick, 2008 ▸); program(s) used to refine structure: *SHELXL97* (Sheldrick, 2008 ▸); molecular graphics: *SHELXTL* (Sheldrick, 2008 ▸); software used to prepare material for publication: *SHELXTL*.

## Supplementary Material

Crystal structure: contains datablock(s) I, global. DOI: 10.1107/S2056989015006933/hb7382sup1.cif

**Fig. 1.**
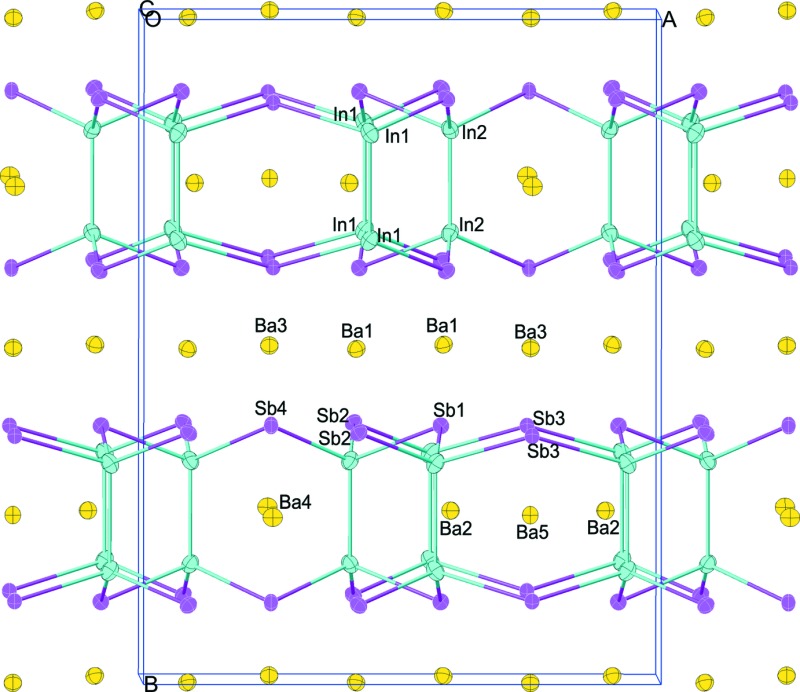
The ellipsoid of Ba5In4Sb6, viewed along the c-axis. The barium cations, Indium atoms and antimony anions were plotted as yellow, light blue and purple spheres, respectively. Ellipsoids are drawn at the 80% probability level.

Structure factors: contains datablock(s) I. DOI: 10.1107/S2056989015006933/hb7382Isup2.hkl

c . DOI: 10.1107/S2056989015006933/hb7382fig1.tif
The ellipsoid of Ba5In4Sb6, viewed along the *c*-axis. The barium cations, Indium atoms and anti­mony anions were plotted as yellow, light blue and purple spheres, respectively. Ellipsoids are drawn at the 80% probability level.

CCDC reference: 1058152

Additional supporting information:  crystallographic information; 3D view; checkCIF report

## supplementary crystallographic information

### S1. Comment

Single crystal of Ba_5_In_4_Sb_6_ was obtained from a high-temperature In-flux reaction and it crystallizes in its own structure type. The layers are constituted by edge-shared In_2_Sb_6_ units and stack along the crystallographic *b*-axis direction. Each In is four-coordinated which is connected by three Sb and the other In atom. All Ba cations are six-coordinated with the bicapped octahedron or triangular prism geometries formed by Sb anions. The bond lengths of In1—In1 and In2—In2 are 2.9655 (11) and 2.8544 (11) Å, respectively, comparable to In—In interconnections in EuIn_2_As_2_ (2.765 Å) (Goforth *et al.*, 2008) and CaIn_2_ (2.92 Å) (Iandelli, 1964).

### S2. Experimental

The title compound was synthesized through the high temperature In flux reaction. All starting elements were handled inside an Argon-filled glove box with controlled oxygen and moisture levels below 0.1 p.p.m.. The reaction conditions were optimized as follows: Ba, In and Sb in a molar ratio of 3:30:5 were loaded in an alumina crucible, which were subsequently flame-sealed in a fused silica tube. The reactants were heated quickly to 1173 K and allowed to dwell at this temperature for 20 h. After a slow cooling process down to 773 K at a rate of 2 K/h, the molten In was removed by centrifugation.

### S3. Refinement

The residual electron densities show a maximum peak of 1.997 e/Å^3^ and a minimum hole of -1.689 e/Å^3^, which are 0.78 and 1.58 Å to Ba3 and In1, respectively.

### Crystal data

**Table:**

Ba_5_In_4_Sb_6_	*F*(000) = 3128
*M**_r_* = 1876.48	*D*_x_ = 5.751 Mg m^−^^3^
Orthorhombic, *P**m**m**n*	Mo *K*α radiation, λ = 0.71073 Å
Hall symbol: -P 2ab 2a	Cell parameters from 1832 reflections
*a* = 14.2723 (13) Å	θ = 2.9–27.6°
*b* = 18.3578 (17) Å	µ = 20.39 mm^−^^1^
*c* = 8.2710 (8) Å	*T* = 120 K
*V* = 2167.1 (4) Å^3^	Block, black
*Z* = 4	0.06 × 0.03 × 0.02 mm

### Data collection

**Table:**

Bruker APEXII CCD diffractometer	2676 independent reflections
Radiation source: fine-focus sealed tube	2098 reflections with *I* > 2σ(*I*)
Graphite monochromator	*R*_int_ = 0.050
φ and ω scans	θ_max_ = 27.6°, θ_min_ = 1.8°
Absorption correction: multi-scan (*SADABS*; Bruker, 2005)	*h* = −18→14
*T*_min_ = 0.374, *T*_max_ = 0.686	*k* = −23→19
12962 measured reflections	*l* = −9→10

### Refinement

**Table:**

Refinement on *F*^2^	Primary atom site location: structure-invariant direct methods
Least-squares matrix: full	Secondary atom site location: difference Fourier map
*R*[*F*^2^ > 2σ(*F*^2^)] = 0.029	*w* = 1/[σ^2^(*F*_o_^2^) + (0.0146*P*)^2^] where *P* = (*F*_o_^2^ + 2*F*_c_^2^)/3
*wR*(*F*^2^) = 0.055	(Δ/σ)_max_ = 0.001
*S* = 1.02	Δρ_max_ = 2.00 e Å^−^^3^
2676 reflections	Δρ_min_ = −1.69 e Å^−^^3^
79 parameters	Extinction correction: *SHELXL97* (Sheldrick, 2008), Fc^*^=kFc[1+0.001xFc^2^λ^3^/sin(2θ)]^-1/4^
0 restraints	Extinction coefficient: 0.000211 (8)

### Special details

**Table:**

Geometry. All e.s.d.'s (except the e.s.d. in the dihedral angle between two l.s. planes) are estimated using the full covariance matrix. The cell e.s.d.'s are taken into account individually in the estimation of e.s.d.'s in distances, angles and torsion angles; correlations between e.s.d.'s in cell parameters are only used when they are defined by crystal symmetry. An approximate (isotropic) treatment of cell e.s.d.'s is used for estimating e.s.d.'s involving l.s. planes.
Refinement. Refinement of *F*^2^ against ALL reflections. The weighted *R*-factor *wR* and goodness of fit *S* are based on *F*^2^, conventional *R*-factors *R* are based on *F*, with *F* set to zero for negative *F*^2^. The threshold expression of *F*^2^ > σ(*F*^2^) is used only for calculating *R*-factors(gt) *etc*. and is not relevant to the choice of reflections for refinement. *R*-factors based on *F*^2^ are statistically about twice as large as those based on *F*, and *R*- factors based on ALL data will be even larger.

### Fractional atomic coordinates and isotropic or equivalent isotropic displacement parameters (Å^2^)

**Table:**

	*x*	*y*	*z*	*U*_iso_*/*U*_eq_	
Ba1	0.08681 (3)	0.00085 (2)	0.23996 (5)	0.00924 (11)	
Ba2	0.10037 (4)	0.2500	0.26369 (7)	0.00879 (14)	
Ba3	0.2500	0.50189 (3)	0.73898 (7)	0.00933 (14)	
Ba4	0.2500	0.7500	0.07159 (11)	0.0113 (2)	
Ba5	0.2500	0.2500	0.70687 (10)	0.00876 (19)	
In1	0.56473 (4)	0.66923 (3)	0.10245 (6)	0.00979 (13)	
In2	0.59568 (3)	0.17226 (3)	0.43328 (6)	0.00990 (13)	
Sb1	0.07830 (3)	0.11748 (3)	0.57615 (6)	0.00809 (11)	
Sb2	0.58565 (3)	0.12044 (3)	0.09457 (6)	0.00865 (12)	
Sb3	0.2500	0.12590 (4)	0.05576 (8)	0.00786 (15)	
Sb4	0.2500	0.61680 (4)	0.39782 (9)	0.00841 (15)	

### Atomic displacement parameters (Å^2^)

**Table:**

	*U*^11^	*U*^22^	*U*^33^	*U*^12^	*U*^13^	*U*^23^
Ba1	0.0093 (2)	0.0090 (2)	0.0094 (2)	0.00006 (17)	−0.00107 (17)	0.00072 (18)
Ba2	0.0094 (3)	0.0092 (3)	0.0078 (3)	0.000	0.0016 (2)	0.000
Ba3	0.0094 (3)	0.0093 (3)	0.0093 (3)	0.000	0.000	0.0005 (2)
Ba4	0.0116 (5)	0.0101 (5)	0.0122 (5)	0.000	0.000	0.000
Ba5	0.0076 (5)	0.0095 (4)	0.0092 (5)	0.000	0.000	0.000
In1	0.0105 (3)	0.0111 (3)	0.0077 (3)	−0.0025 (2)	−0.0016 (2)	0.0011 (2)
In2	0.0088 (3)	0.0102 (3)	0.0107 (3)	0.0017 (2)	−0.0008 (2)	0.0008 (2)
Sb1	0.0087 (3)	0.0088 (3)	0.0068 (2)	0.0004 (2)	−0.00068 (19)	−0.00060 (18)
Sb2	0.0086 (3)	0.0092 (3)	0.0081 (3)	0.0012 (2)	−0.00116 (19)	−0.00030 (19)
Sb3	0.0068 (4)	0.0081 (4)	0.0087 (4)	0.000	0.000	−0.0002 (3)
Sb4	0.0063 (4)	0.0096 (4)	0.0093 (4)	0.000	0.000	−0.0013 (3)

### Geometric parameters (Å, º)

**Table:**

Ba1—Sb4^i^	3.4343 (7)	Ba5—In1^xviii^	3.4173 (7)
Ba1—Sb2^ii^	3.5105 (7)	Ba5—In1^xix^	3.4173 (7)
Ba1—Sb1	3.5115 (7)	Ba5—In1^xx^	3.4173 (7)
Ba1—Sb1^iii^	3.5475 (7)	Ba5—Sb1	3.6184 (6)
Ba1—Sb2^iv^	3.5516 (7)	Ba5—Sb1^i^	3.6184 (6)
Ba1—Sb3	3.6077 (7)	Ba5—Sb1^ix^	3.6184 (6)
Ba1—In1^i^	3.9649 (7)	Ba5—Sb1^ii^	3.6184 (6)
Ba1—Ba2	4.5821 (6)	Ba5—Sb3^xii^	3.6766 (10)
Ba1—Ba1^ii^	4.6582 (10)	Ba5—Sb3^xxi^	3.6766 (10)
Ba1—Ba1^v^	4.6795 (9)	Ba5—Ba2^i^	4.2423 (10)
Ba1—Ba3^i^	4.7394 (8)	In1—Sb1^xxii^	2.8296 (8)
Ba1—Ba3^vi^	4.7537 (8)	In1—Sb2^xv^	2.8398 (7)
Ba2—In1^vii^	3.4100 (8)	In1—In1^xxiii^	2.9655 (11)
Ba2—In1^viii^	3.4100 (8)	In1—Sb3^xxiv^	3.0556 (7)
Ba2—In2^ii^	3.4400 (8)	In1—Ba2^xxiv^	3.4100 (8)
Ba2—In2^i^	3.4400 (8)	In1—Ba5^xvii^	3.4173 (7)
Ba2—Sb1^ix^	3.5632 (7)	In1—Ba1^i^	3.9649 (7)
Ba2—Sb1	3.5632 (7)	In2—Sb4^xxv^	2.7998 (7)
Ba2—Sb3^i^	3.5649 (7)	In2—In2^ix^	2.8544 (11)
Ba2—Sb3	3.5649 (7)	In2—Sb1^ii^	2.9279 (7)
Ba2—Sb2^ii^	3.8291 (7)	In2—Sb2	2.9620 (8)
Ba2—Sb2^i^	3.8291 (7)	In2—Ba2^i^	3.4400 (8)
Ba2—Ba5	4.2423 (10)	In2—Ba3^xxv^	4.0820 (8)
Ba2—Ba2^i^	4.2711 (13)	Sb1—In1^xx^	2.8296 (8)
Ba3—Sb2^x^	3.4833 (7)	Sb1—In2^ii^	2.9279 (7)
Ba3—Sb2^xi^	3.4833 (7)	Sb1—Ba1^iii^	3.5475 (7)
Ba3—Sb3^xii^	3.5169 (9)	Sb1—Ba3^i^	3.5527 (7)
Ba3—Sb4	3.5230 (9)	Sb2—In1^xxvi^	2.8398 (7)
Ba3—Sb1^ix^	3.5527 (7)	Sb2—Ba3^xxv^	3.4833 (7)
Ba3—Sb1^i^	3.5527 (7)	Sb2—Ba1^ii^	3.5105 (7)
Ba3—In2^x^	4.0820 (8)	Sb2—Ba1^xxvii^	3.5516 (7)
Ba3—In2^xi^	4.0820 (8)	Sb2—Ba4^xiv^	3.6122 (6)
Ba3—Ba5	4.6318 (7)	Sb2—Ba2^i^	3.8291 (7)
Ba3—Ba1^ix^	4.7394 (8)	Sb3—In1^vii^	3.0556 (7)
Ba3—Ba1^i^	4.7394 (8)	Sb3—In1^xxvi^	3.0556 (7)
Ba3—Ba1^xii^	4.7537 (8)	Sb3—Ba3^vi^	3.5169 (9)
Ba4—Sb2^xiii^	3.6122 (6)	Sb3—Ba2^i^	3.5649 (7)
Ba4—Sb2^xiv^	3.6122 (6)	Sb3—Ba1^ii^	3.6077 (7)
Ba4—Sb2^viii^	3.6122 (6)	Sb3—Ba5^xxviii^	3.6766 (10)
Ba4—Sb2^xv^	3.6122 (6)	Sb4—In2^x^	2.7998 (7)
Ba4—Sb4^xvi^	3.6414 (10)	Sb4—In2^xi^	2.7998 (7)
Ba4—Sb4	3.6414 (10)	Sb4—Ba1^ix^	3.4343 (7)
Ba5—In1^xvii^	3.4173 (7)	Sb4—Ba1^i^	3.4343 (7)
			
Sb4^i^—Ba1—Sb2^ii^	177.466 (19)	Sb2^xiv^—Ba4—Sb2^viii^	80.989 (19)
Sb4^i^—Ba1—Sb1	96.078 (18)	Sb2^xiii^—Ba4—Sb2^xv^	80.989 (19)
Sb2^ii^—Ba1—Sb1	82.282 (15)	Sb2^xiv^—Ba4—Sb2^xv^	82.364 (19)
Sb4^i^—Ba1—Sb1^iii^	84.397 (16)	Sb2^viii^—Ba4—Sb2^xv^	135.28 (3)
Sb2^ii^—Ba1—Sb1^iii^	93.674 (16)	Sb2^xiii^—Ba4—Sb4^xvi^	136.392 (16)
Sb1—Ba1—Sb1^iii^	90.625 (16)	Sb2^xiv^—Ba4—Sb4^xvi^	80.779 (13)
Sb4^i^—Ba1—Sb2^iv^	84.553 (18)	Sb2^viii^—Ba4—Sb4^xvi^	80.780 (13)
Sb2^ii^—Ba1—Sb2^iv^	97.002 (15)	Sb2^xv^—Ba4—Sb4^xvi^	136.392 (16)
Sb1—Ba1—Sb2^iv^	177.436 (18)	Sb2^xiii^—Ba4—Sb4	80.780 (13)
Sb1^iii^—Ba1—Sb2^iv^	86.962 (15)	Sb2^xiv^—Ba4—Sb4	136.392 (16)
Sb4^i^—Ba1—Sb3	97.059 (16)	Sb2^viii^—Ba4—Sb4	136.392 (16)
Sb2^ii^—Ba1—Sb3	84.841 (15)	Sb2^xv^—Ba4—Sb4	80.779 (13)
Sb1—Ba1—Sb3	88.211 (17)	Sb4^xvi^—Ba4—Sb4	84.37 (3)
Sb1^iii^—Ba1—Sb3	178.223 (18)	In1^xvii^—Ba5—In1^xviii^	101.39 (2)
Sb2^iv^—Ba1—Sb3	94.182 (17)	In1^xvii^—Ba5—In1^xix^	51.430 (19)
Sb4^i^—Ba1—In1^i^	89.069 (17)	In1^xviii^—Ba5—In1^xix^	125.03 (3)
Sb2^ii^—Ba1—In1^i^	90.672 (16)	In1^xvii^—Ba5—In1^xx^	125.03 (3)
Sb1—Ba1—In1^i^	133.506 (16)	In1^xviii^—Ba5—In1^xx^	51.430 (19)
Sb1^iii^—Ba1—In1^i^	43.815 (13)	In1^xix^—Ba5—In1^xx^	101.39 (2)
Sb2^iv^—Ba1—In1^i^	43.967 (12)	In1^xvii^—Ba5—Sb1	162.493 (19)
Sb3—Ba1—In1^i^	137.062 (18)	In1^xviii^—Ba5—Sb1	84.581 (13)
Sb4^i^—Ba1—Ba2	125.651 (17)	In1^xix^—Ba5—Sb1	111.730 (13)
Sb2^ii^—Ba1—Ba2	54.554 (13)	In1^xx^—Ba5—Sb1	47.322 (13)
Sb1—Ba1—Ba2	50.129 (13)	In1^xvii^—Ba5—Sb1^i^	47.322 (13)
Sb1^iii^—Ba1—Ba2	128.386 (16)	In1^xviii^—Ba5—Sb1^i^	111.730 (13)
Sb2^iv^—Ba1—Ba2	131.250 (16)	In1^xix^—Ba5—Sb1^i^	84.581 (13)
Sb3—Ba1—Ba2	49.885 (14)	In1^xx^—Ba5—Sb1^i^	162.493 (19)
In1^i^—Ba1—Ba2	145.225 (16)	Sb1—Ba5—Sb1^i^	145.23 (3)
Sb4^i^—Ba1—Ba1^ii^	47.298 (11)	In1^xvii^—Ba5—Sb1^ix^	111.730 (13)
Sb2^ii^—Ba1—Ba1^ii^	134.518 (11)	In1^xviii^—Ba5—Sb1^ix^	47.322 (13)
Sb1—Ba1—Ba1^ii^	91.983 (11)	In1^xix^—Ba5—Sb1^ix^	162.493 (19)
Sb1^iii^—Ba1—Ba1^ii^	131.624 (11)	In1^xx^—Ba5—Sb1^ix^	84.581 (13)
Sb2^iv^—Ba1—Ba1^ii^	90.267 (10)	Sb1—Ba5—Sb1^ix^	84.494 (18)
Sb3—Ba1—Ba1^ii^	49.790 (10)	Sb1^i^—Ba5—Sb1^ix^	85.261 (18)
In1^i^—Ba1—Ba1^ii^	123.058 (10)	In1^xvii^—Ba5—Sb1^ii^	84.581 (13)
Ba2—Ba1—Ba1^ii^	87.579 (9)	In1^xviii^—Ba5—Sb1^ii^	162.493 (19)
Sb4^i^—Ba1—Ba1^v^	132.64 (2)	In1^xix^—Ba5—Sb1^ii^	47.322 (13)
Sb2^ii^—Ba1—Ba1^v^	48.878 (11)	In1^xx^—Ba5—Sb1^ii^	111.730 (13)
Sb1—Ba1—Ba1^v^	131.107 (18)	Sb1—Ba5—Sb1^ii^	85.261 (18)
Sb1^iii^—Ba1—Ba1^v^	90.449 (16)	Sb1^i^—Ba5—Sb1^ii^	84.494 (18)
Sb2^iv^—Ba1—Ba1^v^	48.124 (12)	Sb1^ix^—Ba5—Sb1^ii^	145.23 (3)
Sb3—Ba1—Ba1^v^	89.306 (16)	In1^xvii^—Ba5—Sb3^xii^	50.868 (13)
In1^i^—Ba1—Ba1^v^	57.489 (12)	In1^xviii^—Ba5—Sb3^xii^	50.868 (13)
Ba2—Ba1—Ba1^v^	93.748 (14)	In1^xix^—Ba5—Sb3^xii^	84.64 (2)
Ba1^ii^—Ba1—Ba1^v^	121.973 (11)	In1^xx^—Ba5—Sb3^xii^	84.64 (2)
Sb4^i^—Ba1—Ba3^i^	47.852 (14)	Sb1—Ba5—Sb3^xii^	130.627 (17)
Sb2^ii^—Ba1—Ba3^i^	130.506 (15)	Sb1^i^—Ba5—Sb3^xii^	79.508 (13)
Sb1—Ba1—Ba3^i^	48.233 (12)	Sb1^ix^—Ba5—Sb3^xii^	79.508 (13)
Sb1^iii^—Ba1—Ba3^i^	86.937 (14)	Sb1^ii^—Ba5—Sb3^xii^	130.627 (17)
Sb2^iv^—Ba1—Ba3^i^	132.382 (15)	In1^xvii^—Ba5—Sb3^xxi^	84.64 (2)
Sb3—Ba1—Ba3^i^	93.283 (15)	In1^xviii^—Ba5—Sb3^xxi^	84.64 (2)
In1^i^—Ba1—Ba3^i^	120.632 (14)	In1^xix^—Ba5—Sb3^xxi^	50.868 (13)
Ba2—Ba1—Ba3^i^	87.281 (13)	In1^xx^—Ba5—Sb3^xxi^	50.868 (13)
Ba1^ii^—Ba1—Ba3^i^	60.565 (7)	Sb1—Ba5—Sb3^xxi^	79.508 (13)
Ba1^v^—Ba1—Ba3^i^	177.275 (17)	Sb1^i^—Ba5—Sb3^xxi^	130.627 (17)
Sb4^i^—Ba1—Ba3^vi^	89.567 (16)	Sb1^ix^—Ba5—Sb3^xxi^	130.627 (17)
Sb2^ii^—Ba1—Ba3^vi^	92.956 (14)	Sb1^ii^—Ba5—Sb3^xxi^	79.508 (13)
Sb1—Ba1—Ba3^vi^	135.534 (16)	Sb3^xii^—Ba5—Sb3^xxi^	76.58 (3)
Sb1^iii^—Ba1—Ba3^vi^	133.841 (15)	In1^xvii^—Ba5—Ba2	142.027 (19)
Sb2^iv^—Ba1—Ba3^vi^	46.886 (12)	In1^xviii^—Ba5—Ba2	90.531 (15)
Sb3—Ba1—Ba3^vi^	47.337 (14)	In1^xix^—Ba5—Ba2	142.027 (19)
In1^i^—Ba1—Ba3^vi^	90.508 (13)	In1^xx^—Ba5—Ba2	90.531 (15)
Ba2—Ba1—Ba3^vi^	91.559 (13)	Sb1—Ba5—Ba2	53.194 (13)
Ba1^ii^—Ba1—Ba3^vi^	60.662 (7)	Sb1^i^—Ba5—Ba2	94.75 (2)
Ba1^v^—Ba1—Ba3^vi^	61.312 (12)	Sb1^ix^—Ba5—Ba2	53.194 (13)
Ba3^i^—Ba1—Ba3^vi^	121.212 (11)	Sb1^ii^—Ba5—Ba2	94.75 (2)
In1^vii^—Ba2—In1^viii^	51.55 (2)	Sb3^xii^—Ba5—Ba2	132.701 (10)
In1^vii^—Ba2—In2^ii^	93.462 (18)	Sb3^xxi^—Ba5—Ba2	132.701 (10)
In1^viii^—Ba2—In2^ii^	114.91 (2)	In1^xvii^—Ba5—Ba2^i^	90.531 (15)
In1^vii^—Ba2—In2^i^	114.91 (2)	In1^xviii^—Ba5—Ba2^i^	142.027 (19)
In1^viii^—Ba2—In2^i^	93.462 (18)	In1^xix^—Ba5—Ba2^i^	90.531 (15)
In2^ii^—Ba2—In2^i^	49.02 (2)	In1^xx^—Ba5—Ba2^i^	142.027 (19)
In1^vii^—Ba2—Sb1^ix^	158.09 (2)	Sb1—Ba5—Ba2^i^	94.75 (2)
In1^viii^—Ba2—Sb1^ix^	109.515 (14)	Sb1^i^—Ba5—Ba2^i^	53.194 (13)
In2^ii^—Ba2—Sb1^ix^	85.158 (18)	Sb1^ix^—Ba5—Ba2^i^	94.75 (2)
In2^i^—Ba2—Sb1^ix^	49.389 (14)	Sb1^ii^—Ba5—Ba2^i^	53.194 (13)
In1^vii^—Ba2—Sb1	109.515 (14)	Sb3^xii^—Ba5—Ba2^i^	132.701 (10)
In1^viii^—Ba2—Sb1	158.09 (2)	Sb3^xxi^—Ba5—Ba2^i^	132.701 (10)
In2^ii^—Ba2—Sb1	49.389 (14)	Ba2—Ba5—Ba2^i^	60.45 (2)
In2^i^—Ba2—Sb1	85.158 (18)	Sb1^xxii^—In1—Sb2^xv^	119.01 (2)
Sb1^ix^—Ba2—Sb1	86.12 (2)	Sb1^xxii^—In1—In1^xxiii^	109.617 (14)
In1^vii^—Ba2—Sb3^i^	86.49 (2)	Sb2^xv^—In1—In1^xxiii^	108.387 (14)
In1^viii^—Ba2—Sb3^i^	51.906 (14)	Sb1^xxii^—In1—Sb3^xxiv^	104.82 (2)
In2^ii^—Ba2—Sb3^i^	161.643 (18)	Sb2^xv^—In1—Sb3^xxiv^	109.04 (2)
In2^i^—Ba2—Sb3^i^	114.761 (14)	In1^xxiii^—In1—Sb3^xxiv^	105.090 (16)
Sb1^ix^—Ba2—Sb3^i^	88.083 (16)	Sb1^xxii^—In1—Ba2^xxiv^	165.95 (2)
Sb1—Ba2—Sb3^i^	147.05 (2)	Sb2^xv^—In1—Ba2^xxiv^	74.947 (19)
In1^vii^—Ba2—Sb3	51.906 (14)	In1^xxiii^—In1—Ba2^xxiv^	64.226 (10)
In1^viii^—Ba2—Sb3	86.49 (2)	Sb3^xxiv^—In1—Ba2^xxiv^	66.660 (19)
In2^ii^—Ba2—Sb3	114.761 (14)	Sb1^xxii^—In1—Ba5^xvii^	70.071 (19)
In2^i^—Ba2—Sb3	161.643 (18)	Sb2^xv^—In1—Ba5^xvii^	170.58 (2)
Sb1^ix^—Ba2—Sb3	147.05 (2)	In1^xxiii^—In1—Ba5^xvii^	64.285 (10)
Sb1—Ba2—Sb3	88.083 (16)	Sb3^xxiv^—In1—Ba5^xvii^	68.962 (19)
Sb3^i^—Ba2—Sb3	79.45 (2)	Ba2^xxiv^—In1—Ba5^xvii^	96.07 (2)
In1^vii^—Ba2—Sb2^ii^	45.739 (13)	Sb1^xxii^—In1—Ba1^i^	60.226 (15)
In1^viii^—Ba2—Sb2^ii^	80.925 (18)	Sb2^xv^—In1—Ba1^i^	60.260 (15)
In2^ii^—Ba2—Sb2^ii^	47.723 (14)	In1^xxiii^—In1—Ba1^i^	141.952 (10)
In2^i^—Ba2—Sb2^ii^	80.950 (18)	Sb3^xxiv^—In1—Ba1^i^	112.95 (2)
Sb1^ix^—Ba2—Sb2^ii^	128.88 (2)	Ba2^xxiv^—In1—Ba1^i^	132.730 (18)
Sb1—Ba2—Sb2^ii^	77.265 (13)	Ba5^xvii^—In1—Ba1^i^	129.15 (2)
Sb3^i^—Ba2—Sb2^ii^	129.50 (2)	Sb4^xxv^—In2—In2^ix^	111.322 (17)
Sb3—Ba2—Sb2^ii^	80.918 (14)	Sb4^xxv^—In2—Sb1^ii^	109.93 (2)
In1^vii^—Ba2—Sb2^i^	80.925 (18)	In2^ix^—In2—Sb1^ii^	110.086 (14)
In1^viii^—Ba2—Sb2^i^	45.739 (13)	Sb4^xxv^—In2—Sb2	113.15 (2)
In2^ii^—Ba2—Sb2^i^	80.950 (18)	In2^ix^—In2—Sb2	108.733 (14)
In2^i^—Ba2—Sb2^i^	47.723 (14)	Sb1^ii^—In2—Sb2	103.32 (2)
Sb1^ix^—Ba2—Sb2^i^	77.265 (13)	Sb4^xxv^—In2—Ba2^i^	173.81 (3)
Sb1—Ba2—Sb2^i^	128.88 (2)	In2^ix^—In2—Ba2^i^	65.488 (10)
Sb3^i^—Ba2—Sb2^i^	80.918 (14)	Sb1^ii^—In2—Ba2^i^	67.497 (17)
Sb3—Ba2—Sb2^i^	129.50 (2)	Sb2—In2—Ba2^i^	73.039 (18)
Sb2^ii^—Ba2—Sb2^i^	76.80 (2)	Sb4^xxv^—In2—Ba3^xxv^	58.068 (19)
In1^vii^—Ba2—Ba5	147.398 (15)	In2^ix^—In2—Ba3^xxv^	140.012 (10)
In1^viii^—Ba2—Ba5	147.398 (15)	Sb1^ii^—In2—Ba3^xxv^	109.592 (19)
In2^ii^—Ba2—Ba5	93.275 (18)	Sb2—In2—Ba3^xxv^	56.626 (15)
In2^i^—Ba2—Ba5	93.275 (18)	Ba2^i^—In2—Ba3^xxv^	127.911 (18)
Sb1^ix^—Ba2—Ba5	54.396 (13)	In1^xx^—Sb1—In2^ii^	101.88 (2)
Sb1—Ba2—Ba5	54.396 (13)	In1^xx^—Sb1—Ba1	161.99 (2)
Sb3^i^—Ba2—Ba5	96.619 (17)	In2^ii^—Sb1—Ba1	85.363 (18)
Sb3—Ba2—Ba5	96.619 (17)	In1^xx^—Sb1—Ba1^iii^	75.959 (17)
Sb2^ii^—Ba2—Ba5	131.656 (13)	In2^ii^—Sb1—Ba1^iii^	79.632 (17)
Sb2^i^—Ba2—Ba5	131.656 (13)	Ba1—Sb1—Ba1^iii^	89.375 (16)
In1^vii^—Ba2—Ba2^i^	98.579 (14)	In1^xx^—Sb1—Ba3^i^	84.155 (19)
In1^viii^—Ba2—Ba2^i^	98.579 (14)	In2^ii^—Sb1—Ba3^i^	161.77 (2)
In2^ii^—Ba2—Ba2^i^	144.428 (13)	Ba1—Sb1—Ba3^i^	84.272 (16)
In2^i^—Ba2—Ba2^i^	144.428 (13)	Ba1^iii^—Sb1—Ba3^i^	85.293 (16)
Sb1^ix^—Ba2—Ba2^i^	95.073 (13)	In1^xx^—Sb1—Ba2	117.27 (2)
Sb1—Ba2—Ba2^i^	95.073 (13)	In2^ii^—Sb1—Ba2	63.114 (17)
Sb3^i^—Ba2—Ba2^i^	53.198 (11)	Ba1—Sb1—Ba2	80.729 (16)
Sb3—Ba2—Ba2^i^	53.198 (11)	Ba1^iii^—Sb1—Ba2	141.977 (19)
Sb2^ii^—Ba2—Ba2^i^	133.895 (11)	Ba3^i^—Sb1—Ba2	129.426 (19)
Sb2^i^—Ba2—Ba2^i^	133.895 (11)	In1^xx^—Sb1—Ba5	62.606 (19)
Ba5—Ba2—Ba2^i^	59.775 (11)	In2^ii^—Sb1—Ba5	117.648 (19)
Sb2^x^—Ba3—Sb2^xi^	84.66 (2)	Ba1—Sb1—Ba5	128.543 (19)
Sb2^x^—Ba3—Sb3^xii^	97.024 (18)	Ba1^iii^—Sb1—Ba5	137.18 (2)
Sb2^xi^—Ba3—Sb3^xii^	97.024 (18)	Ba3^i^—Sb1—Ba5	80.461 (15)
Sb2^x^—Ba3—Sb4	86.702 (17)	Ba2—Sb1—Ba5	72.411 (18)
Sb2^xi^—Ba3—Sb4	86.702 (17)	In1^xxvi^—Sb2—In2	118.55 (2)
Sb3^xii^—Ba3—Sb4	174.94 (2)	In1^xxvi^—Sb2—Ba3^xxv^	158.88 (2)
Sb2^x^—Ba3—Sb1^ix^	178.605 (18)	In2—Sb2—Ba3^xxv^	78.130 (18)
Sb2^xi^—Ba3—Sb1^ix^	94.053 (13)	In1^xxvi^—Sb2—Ba1^ii^	82.178 (18)
Sb3^xii^—Ba3—Sb1^ix^	82.585 (17)	In2—Sb2—Ba1^ii^	84.879 (18)
Sb4—Ba3—Sb1^ix^	93.769 (17)	Ba3^xxv^—Sb2—Ba1^ii^	86.909 (16)
Sb2^x^—Ba3—Sb1^i^	94.053 (13)	In1^xxvi^—Sb2—Ba1^xxvii^	75.772 (18)
Sb2^xi^—Ba3—Sb1^i^	178.605 (18)	In2—Sb2—Ba1^xxvii^	159.71 (2)
Sb3^xii^—Ba3—Sb1^i^	82.585 (17)	Ba3^xxv^—Sb2—Ba1^xxvii^	85.014 (17)
Sb4—Ba3—Sb1^i^	93.769 (17)	Ba1^ii^—Sb2—Ba1^xxvii^	82.998 (15)
Sb1^ix^—Ba3—Sb1^i^	87.23 (2)	In1^xxvi^—Sb2—Ba4^xiv^	93.71 (2)
Sb2^x^—Ba3—In2^x^	45.244 (13)	In2—Sb2—Ba4^xiv^	96.72 (2)
Sb2^xi^—Ba3—In2^x^	91.297 (18)	Ba3^xxv^—Sb2—Ba4^xiv^	97.149 (16)
Sb3^xii^—Ba3—In2^x^	140.454 (14)	Ba1^ii^—Sb2—Ba4^xiv^	175.863 (18)
Sb4—Ba3—In2^x^	42.412 (12)	Ba1^xxvii^—Sb2—Ba4^xiv^	96.509 (19)
Sb1^ix^—Ba3—In2^x^	135.44 (2)	In1^xxvi^—Sb2—Ba2^i^	59.314 (17)
Sb1^i^—Ba3—In2^x^	88.172 (14)	In2—Sb2—Ba2^i^	59.238 (16)
Sb2^x^—Ba3—In2^xi^	91.297 (18)	Ba3^xxv^—Sb2—Ba2^i^	135.28 (2)
Sb2^xi^—Ba3—In2^xi^	45.244 (13)	Ba1^ii^—Sb2—Ba2^i^	77.125 (15)
Sb3^xii^—Ba3—In2^xi^	140.454 (14)	Ba1^xxvii^—Sb2—Ba2^i^	132.629 (18)
Sb4—Ba3—In2^xi^	42.412 (12)	Ba4^xiv^—Sb2—Ba2^i^	100.382 (15)
Sb1^ix^—Ba3—In2^xi^	88.172 (14)	In1^vii^—Sb3—In1^xxvi^	119.85 (3)
Sb1^i^—Ba3—In2^xi^	135.44 (2)	In1^vii^—Sb3—Ba3^vi^	81.640 (18)
In2^x^—Ba3—In2^xi^	65.310 (18)	In1^xxvi^—Sb3—Ba3^vi^	81.641 (18)
Sb2^x^—Ba3—Ba5	130.270 (14)	In1^vii^—Sb3—Ba2^i^	123.96 (2)
Sb2^xi^—Ba3—Ba5	130.270 (14)	In1^xxvi^—Sb3—Ba2^i^	61.434 (16)
Sb3^xii^—Ba3—Ba5	51.447 (18)	Ba3^vi^—Sb3—Ba2^i^	141.785 (14)
Sb4—Ba3—Ba5	123.49 (2)	In1^vii^—Sb3—Ba2	61.434 (16)
Sb1^ix^—Ba3—Ba5	50.390 (12)	In1^xxvi^—Sb3—Ba2	123.96 (2)
Sb1^i^—Ba3—Ba5	50.390 (12)	Ba3^vi^—Sb3—Ba2	141.785 (14)
In2^x^—Ba3—Ba5	138.151 (14)	Ba2^i^—Sb3—Ba2	73.60 (2)
In2^xi^—Ba3—Ba5	138.151 (14)	In1^vii^—Sb3—Ba1	77.750 (14)
Sb2^x^—Ba3—Ba1^ix^	132.982 (18)	In1^xxvi^—Sb3—Ba1	154.85 (2)
Sb2^xi^—Ba3—Ba1^ix^	91.140 (13)	Ba3^vi^—Sb3—Ba1	83.695 (17)
Sb3^xii^—Ba3—Ba1^ix^	129.919 (16)	Ba2^i^—Sb3—Ba1	126.13 (2)
Sb4—Ba3—Ba1^ix^	46.281 (12)	Ba2—Sb3—Ba1	79.407 (14)
Sb1^ix^—Ba3—Ba1^ix^	47.495 (11)	In1^vii^—Sb3—Ba1^ii^	154.85 (2)
Sb1^i^—Ba3—Ba1^ix^	90.133 (16)	In1^xxvi^—Sb3—Ba1^ii^	77.750 (14)
In2^x^—Ba3—Ba1^ix^	88.244 (15)	Ba3^vi^—Sb3—Ba1^ii^	83.695 (17)
In2^xi^—Ba3—Ba1^ix^	55.877 (11)	Ba2^i^—Sb3—Ba1^ii^	79.407 (14)
Ba5—Ba3—Ba1^ix^	86.529 (14)	Ba2—Sb3—Ba1^ii^	126.13 (2)
Sb2^x^—Ba3—Ba1^i^	91.140 (13)	Ba1—Sb3—Ba1^ii^	80.42 (2)
Sb2^xi^—Ba3—Ba1^i^	132.982 (18)	In1^vii^—Sb3—Ba5^xxviii^	60.170 (15)
Sb3^xii^—Ba3—Ba1^i^	129.919 (16)	In1^xxvi^—Sb3—Ba5^xxviii^	60.170 (15)
Sb4—Ba3—Ba1^i^	46.281 (12)	Ba3^vi^—Sb3—Ba5^xxviii^	80.13 (2)
Sb1^ix^—Ba3—Ba1^i^	90.133 (16)	Ba2^i^—Sb3—Ba5^xxviii^	89.005 (18)
Sb1^i^—Ba3—Ba1^i^	47.495 (11)	Ba2—Sb3—Ba5^xxviii^	89.005 (18)
In2^x^—Ba3—Ba1^i^	55.877 (11)	Ba1—Sb3—Ba5^xxviii^	136.530 (13)
In2^xi^—Ba3—Ba1^i^	88.244 (15)	Ba1^ii^—Sb3—Ba5^xxviii^	136.530 (13)
Ba5—Ba3—Ba1^i^	86.529 (14)	In2^x^—Sb4—In2^xi^	103.75 (3)
Ba1^ix^—Ba3—Ba1^i^	58.870 (14)	In2^x^—Sb4—Ba1^ix^	162.15 (3)
Sb2^x^—Ba3—Ba1^xii^	48.100 (12)	In2^xi^—Sb4—Ba1^ix^	83.388 (15)
Sb2^xi^—Ba3—Ba1^xii^	89.545 (17)	In2^x^—Sb4—Ba1^i^	83.388 (15)
Sb3^xii^—Ba3—Ba1^xii^	48.968 (13)	In2^xi^—Sb4—Ba1^i^	162.15 (3)
Sb4—Ba3—Ba1^xii^	134.789 (16)	Ba1^ix^—Sb4—Ba1^i^	85.40 (2)
Sb1^ix^—Ba3—Ba1^xii^	131.442 (18)	In2^x^—Sb4—Ba3	79.52 (2)
Sb1^i^—Ba3—Ba1^xii^	89.193 (13)	In2^xi^—Sb4—Ba3	79.52 (2)
In2^x^—Ba3—Ba1^xii^	92.751 (13)	Ba1^ix^—Sb4—Ba3	85.867 (17)
In2^xi^—Ba3—Ba1^xii^	125.221 (17)	Ba1^i^—Sb4—Ba3	85.867 (17)
Ba5—Ba3—Ba1^xii^	92.258 (15)	In2^x^—Sb4—Ba4	97.21 (2)
Ba1^ix^—Ba3—Ba1^xii^	178.779 (18)	In2^xi^—Sb4—Ba4	97.21 (2)
Ba1^i^—Ba3—Ba1^xii^	121.212 (11)	Ba1^ix^—Sb4—Ba4	98.083 (19)
Sb2^xiii^—Ba4—Sb2^xiv^	135.28 (3)	Ba1^i^—Sb4—Ba4	98.083 (19)
Sb2^xiii^—Ba4—Sb2^viii^	82.364 (19)	Ba3—Sb4—Ba4	174.60 (3)

Symmetry codes: (i) −*x*+1/2, −*y*+1/2, *z*; (ii) −*x*+1/2, *y*, *z*; (iii) −*x*, −*y*, −*z*+1; (iv) *x*−1/2, −*y*, −*z*; (v) −*x*, −*y*, −*z*; (vi) −*x*+1/2, −*y*+1/2, *z*−1; (vii) *x*−1/2, *y*−1/2, −*z*; (viii) *x*−1/2, −*y*+1, −*z*; (ix) *x*, −*y*+1/2, *z*; (x) −*x*+1, *y*+1/2, −*z*+1; (xi) *x*−1/2, *y*+1/2, −*z*+1; (xii) −*x*+1/2, −*y*+1/2, *z*+1; (xiii) *x*−1/2, *y*+1/2, −*z*; (xiv) −*x*+1, −*y*+1, −*z*; (xv) −*x*+1, *y*+1/2, −*z*; (xvi) −*x*+1/2, −*y*+3/2, *z*; (xvii) −*x*+1, −*y*+1, −*z*+1; (xviii) *x*−1/2, −*y*+1, −*z*+1; (xix) −*x*+1, *y*−1/2, −*z*+1; (xx) *x*−1/2, *y*−1/2, −*z*+1; (xxi) *x*, *y*, *z*+1; (xxii) *x*+1/2, *y*+1/2, −*z*+1; (xxiii) *x*, −*y*+3/2, *z*; (xxiv) *x*+1/2, *y*+1/2, −*z*; (xxv) *x*+1/2, *y*−1/2, −*z*+1; (xxvi) −*x*+1, *y*−1/2, −*z*; (xxvii) *x*+1/2, −*y*, −*z*; (xxviii) *x*, *y*, *z*−1.
