# Supplementary figures and images for: Crystal structure of Ba5In4Sb6
Source: Acta Crystallogr E Crystallogr Commun. 2015 Apr 9;71(Pt 5):i4. doi: 10.1107/S2056989015006933 (PMC4420070; doi:10.1107/S2056989015006933)

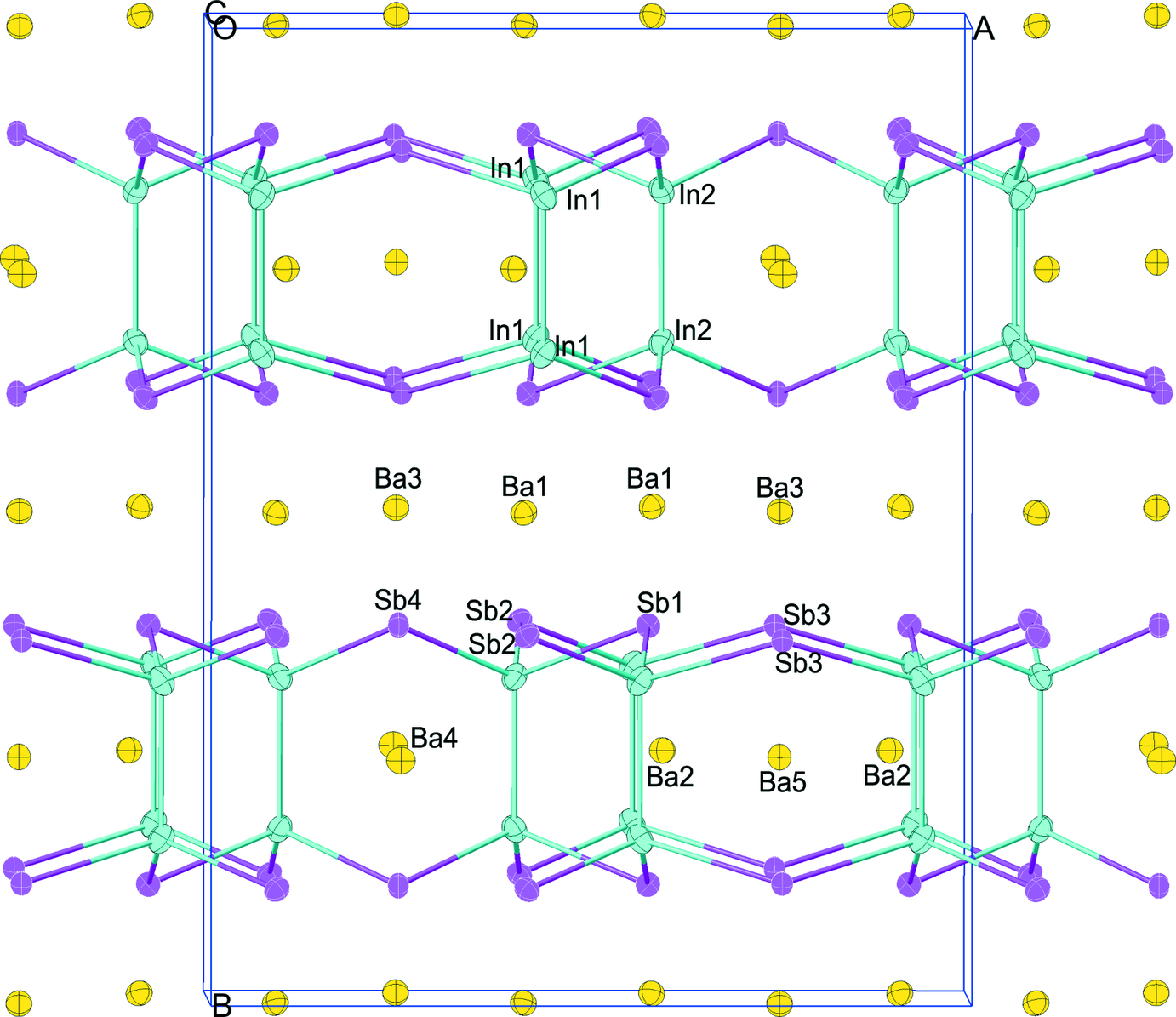

Supplement: Supplementary file 3 [file e-71-000i4-fig1.tif]
